# Resistin-like molecule β acts as a mitogenic factor in hypoxic pulmonary hypertension via the Ca^2+^-dependent PI3K/Akt/mTOR and PKC/MAPK signaling pathways

**DOI:** 10.1186/s12931-020-01598-4

**Published:** 2021-01-06

**Authors:** Heshen Tian, Lei Liu, Ying Wu, Ruiwen Wang, Yongliang Jiang, Ruicheng Hu, Liming Zhu, Linwei Li, Yanyan Fang, Chulan Yang, Lianzhi Ji, Guoyu Liu, Aiguo Dai

**Affiliations:** 1 Department of Respiratory Medicine & Department of Geriatric, Hunan Provincial People’s Hospital/The First Affiliated Hospital of Hunan Normal University, Changsha, 410016 Hunan People’s Republic of China; 2 Department of Respiratory Diseases, Medical School, Hunan University of Chinese Medicine, Changsha, 410208 Hunan People’s Republic of China; 3 State Key Lab of Respiratory Diseases, The First Affiliated Hospital, Guangzhou Medical University, Guangzhou, 510120 Guangdong People’s Republic of China

**Keywords:** Hypoxic pulmonary arterial hypertension, Resistin-like molecule β, Ca^2+^, SOCE, Pulmonary vascular remodeling, Signaling pathway

## Abstract

**Background:**

Pulmonary arterial smooth muscle cell (PASMC) proliferation plays a crucial role in hypoxia-induced pulmonary hypertension (HPH). Previous studies have found that resistin-like molecule β (RELM-β) is upregulated de novo in response to hypoxia in cultured human PASMCs (hPASMCs). RELM-β has been reported to promote hPASMC proliferation and is involved in pulmonary vascular remodeling in patients with PAH. However, the expression pattern, effects, and mechanisms of action of RELM-β in HPH remain unclear.

**Methods:**

We assessed the expression pattern, mitogenetic effect, and mechanism of action of RELM-β in a rat HPH model and in hPASMCs.

**Results:**

Overexpression of RELM-β caused hemodynamic changes in a rat model of HPH similar to those induced by chronic hypoxia, including increased mean right ventricular systolic pressure (mRVSP), right ventricular hypertrophy index (RVHI) and thickening of small pulmonary arterioles. Knockdown of RELM-β partially blocked the increases in mRVSP, RVHI, and vascular remodeling induced by hypoxia. The phosphorylation levels of the PI3K, Akt, mTOR, PKC, and MAPK proteins were significantly up- or downregulated by RELM-β gene overexpression or silencing, respectively. Recombinant RELM-β protein increased the intracellular Ca^2+^ concentration in primary cultured hPASMCs and promoted hPASMC proliferation. The mitogenic effects of RELM-β on hPASMCs and the phosphorylation of PI3K, Akt, mTOR, PKC, and MAPK were suppressed by a Ca^2+^ inhibitor.

**Conclusions:**

Our findings suggest that RELM-β acts as a cytokine-like growth factor in the development of HPH and that the effects of RELM-β are likely to be mediated by the Ca^2+^-dependent PI3K/Akt/mTOR and PKC/MAPK pathways.

## Introduction

Hypoxia-induced pulmonary hypertension (HPH) is a progressive and devastating complication of chronic obstructive pulmonary disease (COPD) that contributes to the morbidity and mortality of patients with various types of lung/heart diseases [1, 2]. Patients with severe COPD often suffer from flow limitation, which leads to alveolar hypoxia and hypoxemia. Sustained alveolar hypoxia induces pulmonary vascular remodeling (HPVR) characterized by abnormal thickening of precapillary pulmonary vessel walls and muscularization of small pulmonary arteries [3, 4]. Abnormal pulmonary arterial smooth muscle cell (PASMC) proliferation is involved in HPVR due to narrowing of the lumen and an increase in the pulmonary circulatory resistance finally resulting in pulmonary arterial hypertension (PAH) [1, 5, 6].

Resistin-like molecule β (RELM-β) belongs to a newly described resistin-like molecule (RELM) gene family that encodes secreted proteins and is the direct ortholog of hypoxia-induced mitogenic factor (HIMF/RELM-α) [7, 8]. RELM-β is inherently expressed in colonic epithelial cells. Hypoxia and inflammation are the main pathological stimulators of RELM-β production in the lung [9–11]. Further studies have proved that high levels of RLEM-β are present in patients with PAH, and RLEM-β overexpression significantly enhances proliferation of transfected hPASMCs [12, 13]. This evidence suggests that RELM-β may play an important role in the HPH development due to its mitogenic effect. However, the expression of RELM-β at various stages of hypoxia and the mechanisms by which RELM-β causes abnormal PASMC proliferation have not been investigated.

Renigunta et al. [12, 14] showed that the proliferative effect of RELM-β can be suppressed by two PI3K (phosphatidylinositol 3-kinase) inhibitors and a protein kinase C (PKC) inhibitor, which was also observed in the case of HIMF in mouse cells [10, 11, 15]. PI3K and PKC are the key enzymes that mediate the proliferation of PASMCs by phosphorylating Akt (protein kinase B) and MAPKs (mitogen-activated protein kinases), respectively [16–18]. In an in vitro experimental study, Lee et al. [19] found that hypoxia induces Ca^2+^-dependent phosphorylation of PI3K/Akt/mTOR and PKC/MAPKs in chicken hepatocytes. The PI3K/Akt and PKC signaling pathways activate cyclins E and D1 and cyclin-dependent kinases (CDK) 2 and 4 and promote cell proliferation and differentiation [20]. A recent study [21] demonstrated that HIMF increases intracellular Ca^2+^ concentration through CaSR (Ca^2+^-sensing receptor) and a SOCE (store-operated calcium entry)-dependent manner and induces PASMC proliferation in PAH. Since RELM-β is known as a functional substitute for HIMF in humans [12], we sought to determine whether RELM-β can increase Ca^2+^ concentration in hPASMCs.

In the present study, the expression pattern of RELM-β in a rat model of HPH was evaluated and the hypothesis that RELM-β plays a role in HPVR and HPH was tested. We also investigated the role of RELM-β in PASMC proliferation and determined whether the mitogenic effect of RELM-β is mediated by the PI3K/Akt/mTOR and PKC/MAPK signaling pathways and whether Ca^2+^ is involved in this process. Our results indicate that RELM-β may be a potential target for HPH therapy.

## Material and methods

### Hypoxia-induced rat PAH model

A total of 60 adult male Sprague–Dawley (SD) rats (260–330 g, 12 weeks old) with matched body weight and ages were randomly divided into the hypoxia and control groups. Hypoxia groups were exposed to normobaric hypoxia (10% oxygen environment) in a ventilated hypoxia chamber for three weeks according to the previous report [22]. Rats used for mRVSP measurement (similar to our previous report) were anesthetized with an i.p. injection of pentobarbital sodium (40 mg/kg) and a s.c. injection of buprenorphine (0.05 mg/kg). All of the rats were euthanized by exsanguination. Bronchoalveolar lavage fluid (BALF) was collected from the right lung (left lung was tied off by hemostatic clips) by tracheal injection with 2.5 ml of sterile saline solution under constant pressure (repeated three times). Recycled BALF was centrifuged, and the supernatant was stored at − 80 °C for use in ELISA. Afterwards, the right lung was tied off, and the left lung was inflated with 4% paraformaldehyde fixing solution (Beyotime, CHN) at a constant pressure (25 cm H_2_O). The heart and lungs were removed en bloc and placed on ice. The right lung was frozen in liquid nitrogen for use in Western blot or RT-PCR. The heart and left lung were immediately placed in 4% paraformaldehyde fixing solution (> 5 volumes of each sample) at 4 °C for 48–72 h fixing followed by paraffin embedding. RVHI was assessed based on the left/right ventricular weight ratio (RS/LV + S), and HPVR was assessed by the morphological analysis of small pulmonary vessels (wall thickness, WT%; wall area, WA%; and lumen area, LA%) by H&E staining. Isolation of intralobar pulmonary arteries was performed as described previously [22, 23]. Small lung arteries (external diameter between 25 and 200 μm) were classified as fully muscular (FM), partially muscular (PM), or nonmuscular (NM) according to the level of smooth muscle actin (α-SMA) staining. The percent media thickness (%MT) was calculated and measured as described [24]. The animals were obtained from the Animal Experimental Center of Hunan University of Chinese Medicine, China.

### Knockdown and overexpression of RELM-β in vivo

Male Sprague–Dawley rats were used in this study. To perform RELM-β gene overexpression and knockdown, an effective short hairpin RNA (shRNAmiR) targeting rat RELM-β was constructed and subcloned into the lentiviral U6-MCS-Ubi-EGFP plasmid. The U6-MCS-Ubi-EGFP-RELM-β or U6-MCS-Ubi-EGFP-RELM-β-shRNAmiR plasmids and lentiviral packaging plasmid were cotransfected into 293T cells (ATCC, Manassas, United States). The culture supernatant was harvested, and the viral titer was determined 48 h after the transfection. Six days before hypoxia exposure, the rats in each group received intratracheal instillation with Lv-EGFP-RELM-β-shRNAmiR or Lv-EGFP-RELM-β plasmids (1.5 × 10^8^ transducing units in a total volume of 150 μl at a daily dose of 50 μl for 3 days) as described previously [25]. The control group was treated with an equal amount of lentiviral (Lv)-null or saline, and other conditions were identical. Frozen sections of the lung were used to visualize EGFP expression as described [25]. After 21 days of exposure to hypoxia or normoxia, the expression of RELM-β mRNA and protein in the rat lung was detected by Western blot and real-time PCR.

### Cell culture and pretreatment

PASMCs were identified by expression of α-actin (≥ 95% of the cells) by immunofluorescence using an antibody against α-SMA (1:200, Abcam, USA). Primary human pulmonary artery SMCs (ScienCell Research Laboratories, USA) were maintained in a Petri dish containing prechilled PBS, 2% penicillin–streptomycin, Ca^2+^-free HBSS, and 0.5% fetal calf serum mixed with 20% DMEM/F12 medium (Billups-Rothenberg, Del Mar, USA). Then, the cells were cultured in an atmosphere containing 5% CO_2_ and 21% O_2_ or 3% O_2_ in a humidified incubator at 37 °C for 3, 6, 12, 24, and 48 h for normoxia or hypoxia treatments, respectively. For cell proliferation, Ca^2+^ imaging, and detection of the phosphorylation signaling pathway, PASMCs were pretreated with cyclopiazonic acid (CPA), nifedipine (Nifed), EGTA (Santa Cruz, USA), BAPTA/AM (Santa Cruz, USA), LY294002 (Santa Cruz, USA), Perifosine (Santa Cruz, USA), LY317615 (Santa Cruz, USA), rapamycin (Santa Cruz, USA), or PD98059 (Santa Cruz, USA) and seeded onto 25 mm glass coverslips as indicated. Then, various concentrations of recombinant human RELM-β protein (0–40 ng/ml, Abgent, USA) were used to stimulate PASMCs for various periods (0–72 h). Cells of passages 3–7 were used in the experiments. When cells in the logarithmic growth phase reached a density of 70%, cell cycle was synchronized by incubation in the serum-free medium for 24 h.

### Cell viability and proliferation assay

Cell viability was determined using a CCK-8 cell counting kit (Beyotime Biotechnology, Jiangsu, China) according to the manufacturer's protocol. After the treatments, 10 μl of CCK-8 solution was added to each well and incubated for 1 h. The absorbance of each well was measured at a wavelength of 450 nm using a microplate reader (BMG LABTECH, Durham, NC, USA). The cell proliferation assay was performed using EdU immunofluorescent staining as described previously [26]. For each EdU experiment, three random fields were imaged at × 20 magnification, and the fraction of EdU-positive cells was calculated as the percentage of the total number of cells in each field.

### H&E staining and immunohistochemistry

Paraffin-embedded rat lung tissue was sectioned, dewaxed, and rehydrated as described previously [22]. Hematoxylin and eosin (H&E) staining was performed according to the operation manual of a hematoxylin and eosin staining kit (Beyotime, CHN). IHC was performed as described [23]. After blocking nonspecific protein binding, the sections were treated with mouse anti-α-SMA (1:500, Boster, Wuhan, CHN), anti-p-PI3K/anti-PI3K (1:100, Abcam, USA), anti-p-Akt/anti-Akt (1:200, Abcam, UK), anti-p-mTOR/anti-beta-actin (1:100, Abcam, UK), anti-p-PKC/anti-PKC (1:250, Cell Signaling, USA), or anti-p-p44/42/anti-p44/42 antibodies (1:250, Cell Signaling, USA) followed by incubation for 2 h with goat anti-rabbit secondary antibodies (HPR, Boster, CHN); then, ABC horseradish peroxidase reagent (Boster, CHN) was added for 30 min at room temperature. The secondary antibody controls (negative control) were incubated in PBS instead of the primary antibody. Specimens were observed under a fluorescent inverted microscope (IX73-A22FL/PH; Olympus Corporation, Japan), and images were captured by using the Image-Pro Plus software (version 6.0). The average optical density (IOD/area) was used as a semiquantitative index for analysis of the positive signals.

### Immunofluorescence

For the immunofluorescence assay, the lung sections were treated with rabbit anti-RELM-β polyclonal antibodies (1:200, Abcam, UK) and mouse anti-α-SMA antibody (1:500) or the corresponding vehicle overnight at 4 °C. Then, the sections were incubated with FITC-conjugated AffiniPure goat anti-mouse IgG (H + L) (1:50, Boster, CHN) (excitation: 495 nm, emission: 525 nm) and Cy3-conjugated AffiniPure goat anti-mouse IgG (H + L) (1:50, Boster, CHN) (excitation: 554 nm, emission: 568 nm) for 45 min at room temperature in the dark. Finally, the sections were washed in PBS and mounted with Vectashield hardset mounting medium with 4′,6-diamidino-2-phenylindole dilactate (DAPI, Vector Laboratories, USA). Specimens were observed under a fluorescent inverted microscope (IX73-A22FL/PH; Olympus Corporation; light source: UHP) attached to a CCD digital camera (512B Cascade, Roper Scientific, Tucson, AZ). Blue (WU) and green filters (WIBA) were used to detect FITC (green) and Cy3 (red) signals, respectively. The objectives used were 20 × and 40 × . Images were captured by using the Image-Pro Plus software (version 6.0) and colorized with Adobe Photoshop CS6 software (Adobe Systems, Inc., San Jose, CA).

### Western blot analysis

PASMCs and isolated pulmonary artery and rat tissue samples were homogenized, and protein electrophoresis and Western blot were performed as described previously [22]. BCA assay was used to estimate the protein concentration. Protein (40 μg) or medium supernatant (40 μl) samples were loaded onto 6–20% polyacrylamide gels (Bio-Rad). After electrophoresis, proteins were transferred to the nitrocellulose membranes (Bio-Rad) and stained with rabbit anti-RELM-β (1:500, Abcam, UK) or antibodies against phosphorylated and nonphosphorylated PI3K, Akt, mTOR, PKC, and MAPK (1:500 or 1:1000, Santa Cruz, USA) followed by incubation with goat anti-rabbit HRP-labeled secondary antibodies (1:3000, Boster, CHN). Following washing, an enhanced chemiluminescence substrate kit (Boster, CHN) was used for chemiluminescent signals detection. Membranes were imaged on autoradiography image system (ProteinSimple FE 1004, San Jose, USA) and quantified by densitometry with image analysis software (ImageJ).

### ELISA of RELM-β

The RELM-β concentrations in the serum prepared from the peripheral blood (PB) and in BALF were determined using an enzyme-linked immunosorbent assay (ELISA) kit from QiMing (Shanghai, China). The assay was carried out according to the manufacturer’s instructions.

### Real-time PCR analysis

To quantify the gene transcripts of RELM-β, an RNeasy mini kit (Qiagen) was used to extract total RNA from the right lower lung lobe of the rats (200 mg), isolated pulmonary artery, and PASMCs following the manufacturer’s protocol. RT-PCR was conducted using a miScript SYBR Green PCR kit (Qiagen Inc., Valencia, CA). The PCR primers were as follows: rat RELM-β, 5′-CTCCCACTGATAGTCCCA-3′ and 5′-CACAGCCATAGCCACAAG-3′ with a 187 bp amplicon; rat GAPDH, 5′-CATCCTGCGTCTGGACCTGG-3′ and 5′-TAAT GTCACG CACGATTTCC-3′; human RELM-β, 5′-CTGTCACTGGCTGTCTTGT-3′ and 5′-TTGGGA CCCTGGTTTCAT TA-3′ with a 182 bp amplicon; and human GAPDH, 5′-AGACAGCC GCATCTTCTTGT -3′ and 5′-TGATGGCAACAATGTCCACT-3′. The 2^−ΔΔCt^ method was used to calculate the relative fold changes.

### Imaging of intracellular Ca^2+^

The intracellular Ca^2+^ assay was performed according to the previously described protocol [26]. Human PASMCs pretreated with 3.5 μmol/L Fura-2-AM (Invitrogen, USA) were incubated for 1 h at 37 °C and washed twice with Ca^2+^-containing buffer or HBSS (Sigma). Coverslips with hPASMCs were mounted onto a closed polycarbonate chamber clamped on a heated aluminum platform (PH-2; Warner Instrument, Hamden, CT, USA) on the stage of an inverted microscope (Leica, Melville, NY, USA). Then, the cells were alternately stimulated with RELM-β, Ca^2+^-containing buffer, or HBSS through a tee irrigation device. The fluorescence was recorded at 12 s intervals over 10 min using a dual-wavelength excitation fluorimeter. Fluorescent emission was acquired at 510 nm from the regions of interest corresponding to single cells after excitation at 340 and 380 nm (shift in excitation wavelength upon Ca^2+^ binding) in 15–30 cells using a xenon arc lamp, interference filters, an electronic shutter, and a × 20 fluorescence objective. The data are expressed as the 340/380 nm fluorescence ratio, which reflects the cytosolic free calcium concentration (proportional to [Ca^2+^]_i_).

### Statistical analysis

The data were analyzed using the SPSS 18.0 statistical software. Data are expressed as the mean ± SD. Significant differences between two independent groups were determined using Student's *t*-test. For multiple groups, the statistical analyses were performed by ANOVA followed by Tukey’s test. P values less than 0.05 were considered significant.

## Results

### Validation of the HPH rat model

**Fig. 1:**
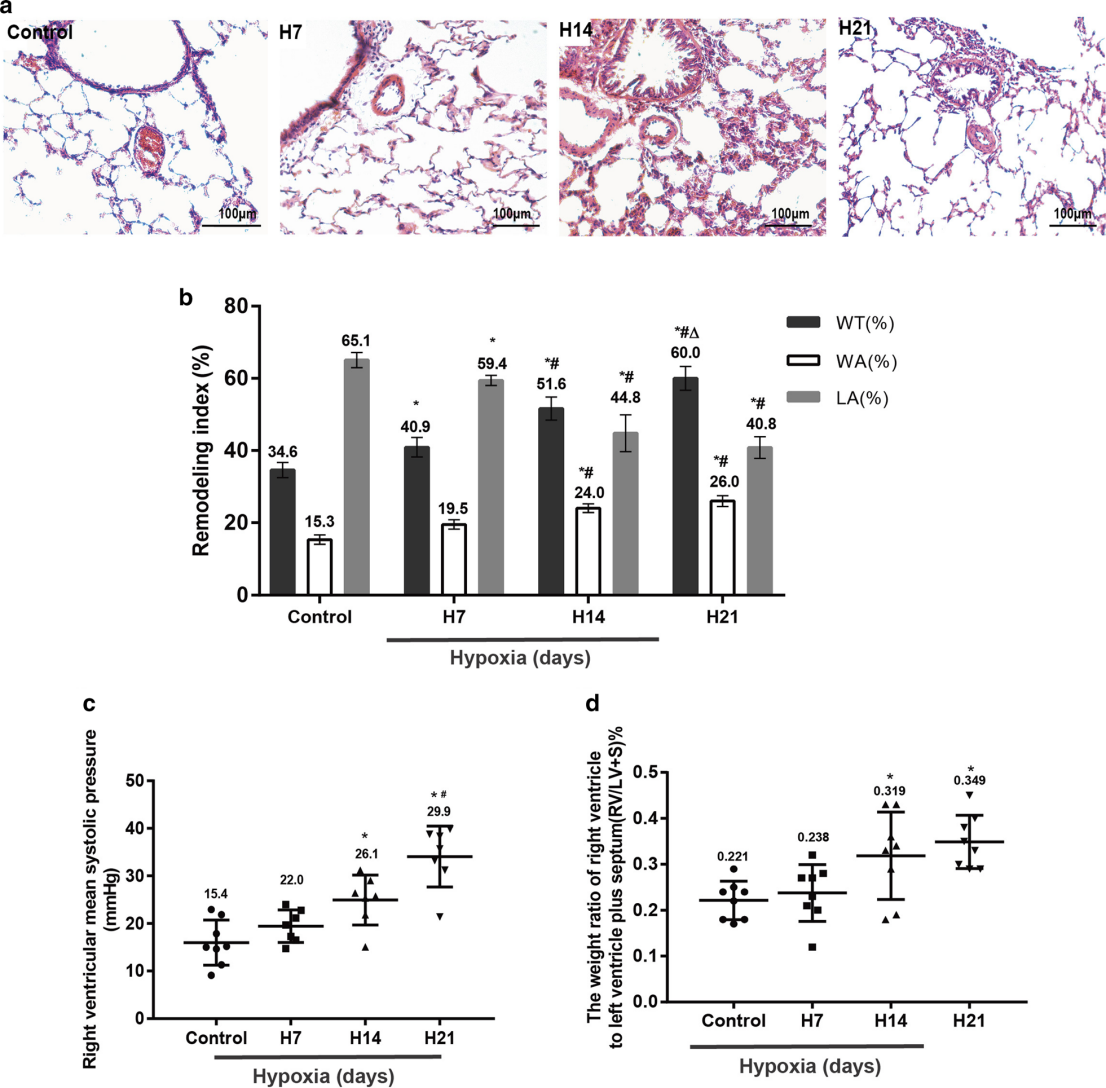
Verification of the hypoxia-induced rat PAH model. The mean arterial wall thickness at different durations of hypoxia (H&E staining, optical microscope, 400 ×) (**a**). Morphometric analysis of rat pulmonary arterioles after exposure to normoxia (Control) or hypoxia for 7 days (H7), 14 days (H14) or 21 days (H21) (**b**). The mRVSP of rats exposed to normoxia or hypoxia for different durations (**c**). Time course of the RV/LV + S ratio in rats exposed to hypoxia (**d**). WT(%), ratio of vascular wall thickness to external diameter; LA(%), ratio of lumen area to total vascular area; WA(%), ratio of vascular wall area to total vascular area; n ≥ 6 in each group. *P < 0.05 versus the control group; ^#^P < 0.05 versus group H7; ^△^P < 0.05 versus group H14

The H&E staining results showed that after hypoxia exposure, the pulmonary artery wall thickness was generally increased and the vascular lumen was narrowed after 7 days of hypoxia (Fig. 1a); these values were maximal at 14 days and remained constant for 21 days of hypoxia; these alterations were statistically significant compared with the parameters of the control group (P < 0.05) (Fig. 1b). Rats manifested profound pulmonary arterial hypertension and right ventricular hypertrophy when examined 21 days after hypoxia induction. Mean PAP (assessed as mRVSP) was measured in conscious rats (mRVSP in normoxic animals was 15.7 ± 1.3 mm Hg). As expected, hypoxic animals developed PH after 7 days of hypoxia (P > 0.05). mRVSP peaked at 14 days and remained at high levels on day 21 after hypoxia induction (P < 0.05) (Fig. 1c). The ratio of RV/(LV + S) in the hypoxia groups showed similar trends (Fig. 1d). These changes in the pulmonary arteries indicated that the model of vascular PAH induced by hypoxia was successfully established.

### Hypoxia enhances RELM-β expression in the rat lung

**Fig. 2:**
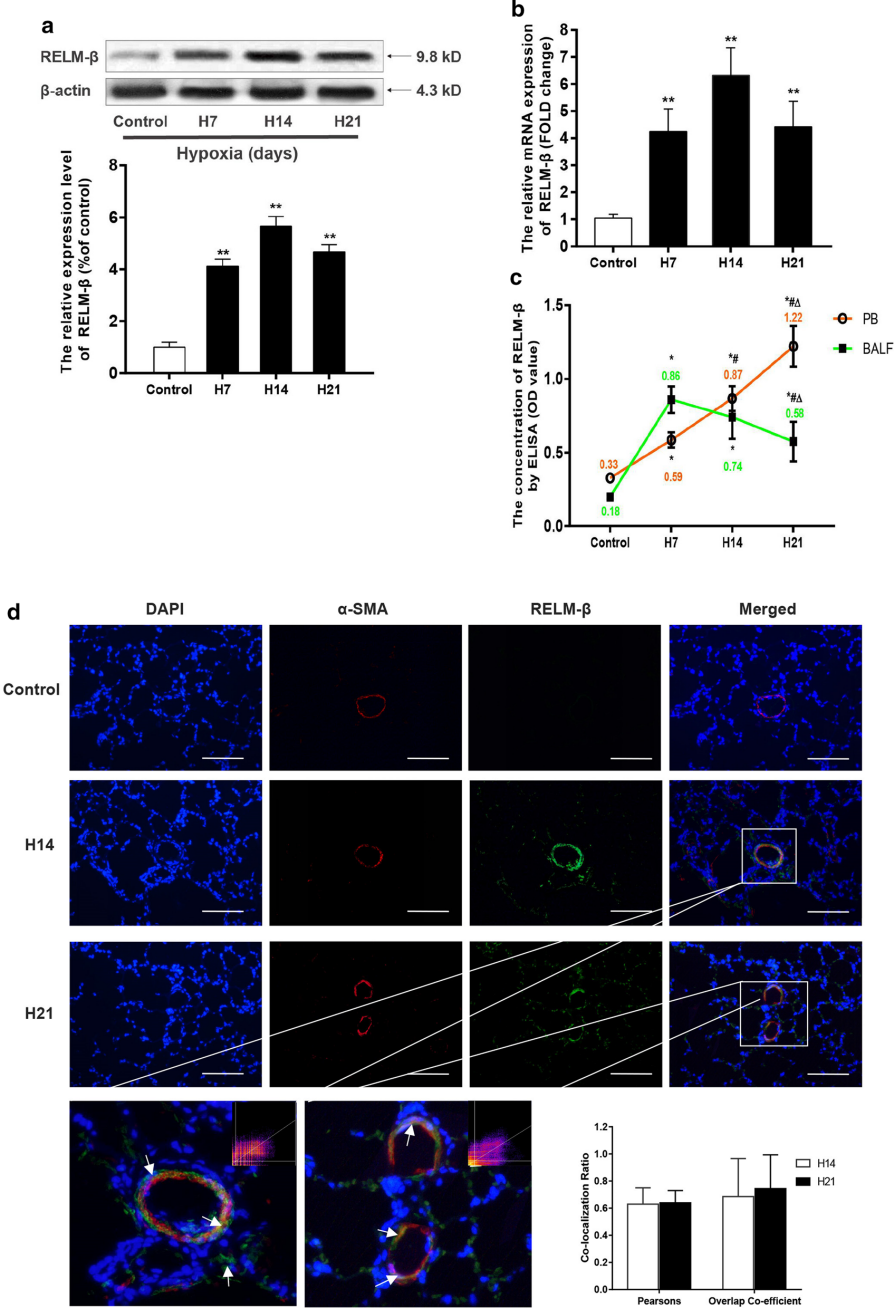
Expression pattern of RELM-β in tissues from an HPH rat model. WB analysis of RELM-β protein expression in rat pulmonary tissue of the control group, H7 group, H14 group and H21 group (**a**). Quantitative real-time PCR (RT-PCR) results of RELM-β mRNA expression in pulmonary artery muscle at different durations of hypoxia (**b**). Enzyme-linked immunosorbent assay (ELISA) analysis showing that the serum concentration of RELM-β protein continued to rise, but in the bronchial alveolar lavage fluid, the concentration showed a trend of first increasing and then decreasing, probably due to its paracrine characteristics and regional differences (**c**). Immunofluorescence colocalization of RELM-β and α-SMA after exposure to hypoxia suggests that RELM-β is expressed in pulmonary arteries (white arrow: pulmonary artery smooth muscle, 400 ×) (**d**). PB, peripheral blood. n ≥ 6 in each group. *P < 0.05 versus the control group; **P < 0.01 versus the control group; ^#^P < 0.05 versus group H7; ^△^P < 0.05 versus group H14

Previous studies using an asthma model and a pulmonary fibrosis model in rodents have shown that RELM-β has inducible properties [13, 27]. In the present study, we estimated the expression pattern of RELM-β in a rat HPH model for the first time. In the normoxia group, RELM-β immunoreactivity was very weak but was significantly increased after 7 days of hypoxia in the pulmonary artery media compared with that in the control group (P < 0.01), peaking after 14 days of hypoxia. Then, the RELM-β protein level was reduced but remained higher than that in the normoxia group (P < 0.01) and the 7-day hypoxia group (P < 0.05) (Fig. 2a). A similar trend was detected in RELM-β mRNA expression assessed by RT-PCR (P < 0.01) (Fig. 2b). RELM-β concentrations in the peripheral serum (PB) and BALF were assayed (see Fig. 2c). Additionally, the lung sections had strong RELM-β immunofluorescence colocalized with α-SMA in the smooth muscle of the pulmonary artery (Fig. 2d). Our results suggest that hypoxia induces sustained RELM-β expression in rat pulmonary artery and lung for at least 21 days.

### Effects of RELM-β on HPH and cardiac and pulmonary artery remodeling

**Fig. 3:**
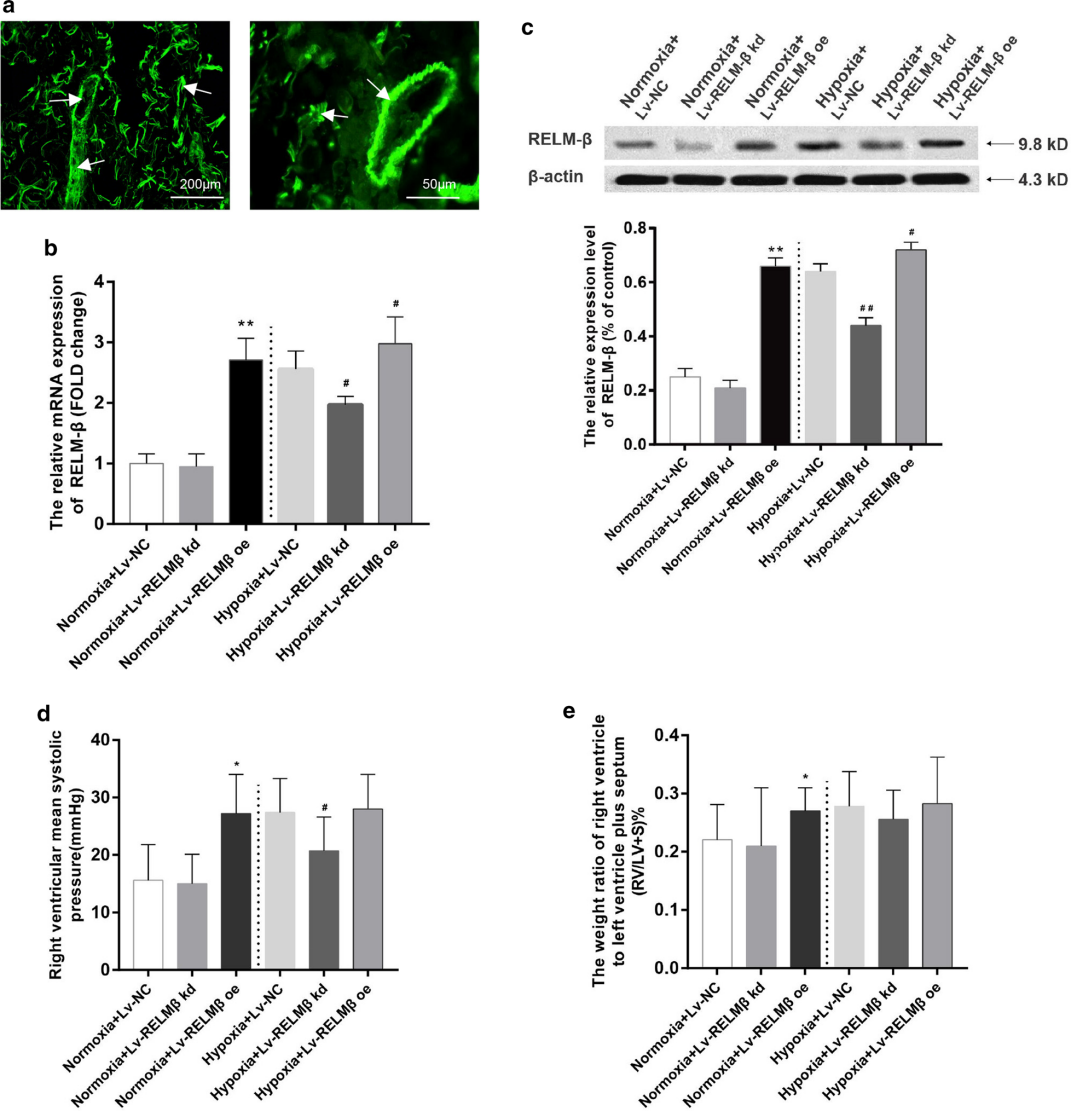
Effects of RELM-β on HPH and cardiac and pulmonary artery remodeling. Rat pulmonary tissues were observed over 21 days after intratracheal gene transfer with Lenti6.3-EGFP (1.5 × 10^8^ transducing units in a total volume of 150 μl at a daily dose of 50 μl for 3 days). The transfection efficiency was indicated by the green fluorescence in the lung tissues (the white arrows show that EGFP was abundantly expressed in lung tissues and blood vessels) (**a**). The relative mRNA level of RELM-β in different treatment groups (**b**). The WB results of RELM-β and the relative protein expression level of RELM-β in different treatment groups (**c**). The mean pulmonary arterial pressure of rats in different groups (**d**). The RV/LV + S ratio in rats in different groups (**e**). n = 6 rats in each group. *P < 0.05 versus the Normoxia + Lv-NC group; **P < 0.01 versus the Hypoxia + Lv-NC group

RELM-β is a secreted protein that has a proliferative effect on PASMCs [12, 28]. To verify the role of RELM-β, gene transfer and silencing of RELM-β were performed in rat lungs. After treatment with Lv-null, Lv-RELM-β, or Lv-RELM-β-RNAi viruses via intratracheal injection, the rats were exposed to normoxia or hypoxia for three weeks. Frozen lung sections from Lv-EGPF-injected rats displayed strong green fluorescence in the lung and pulmonary artery wall confirming that the plasmids were correctly delivered to the targets (Fig. 3a). The results showed that the mRNA and protein levels of RELM-β in the overexpression group were higher compared with those in the Lv-NC and Lv-RELM-β knockdown (kd) groups under the normoxic or hypoxic conditions (P < 0.05). Introduction of Lv-RELM-β-RNAi reduced RELM-β upregulation by hypoxia (P < 0.05), whereas the Lv-null virus did not influence RELM-β expression (Fig. 3b, c).

**Fig. 4:**
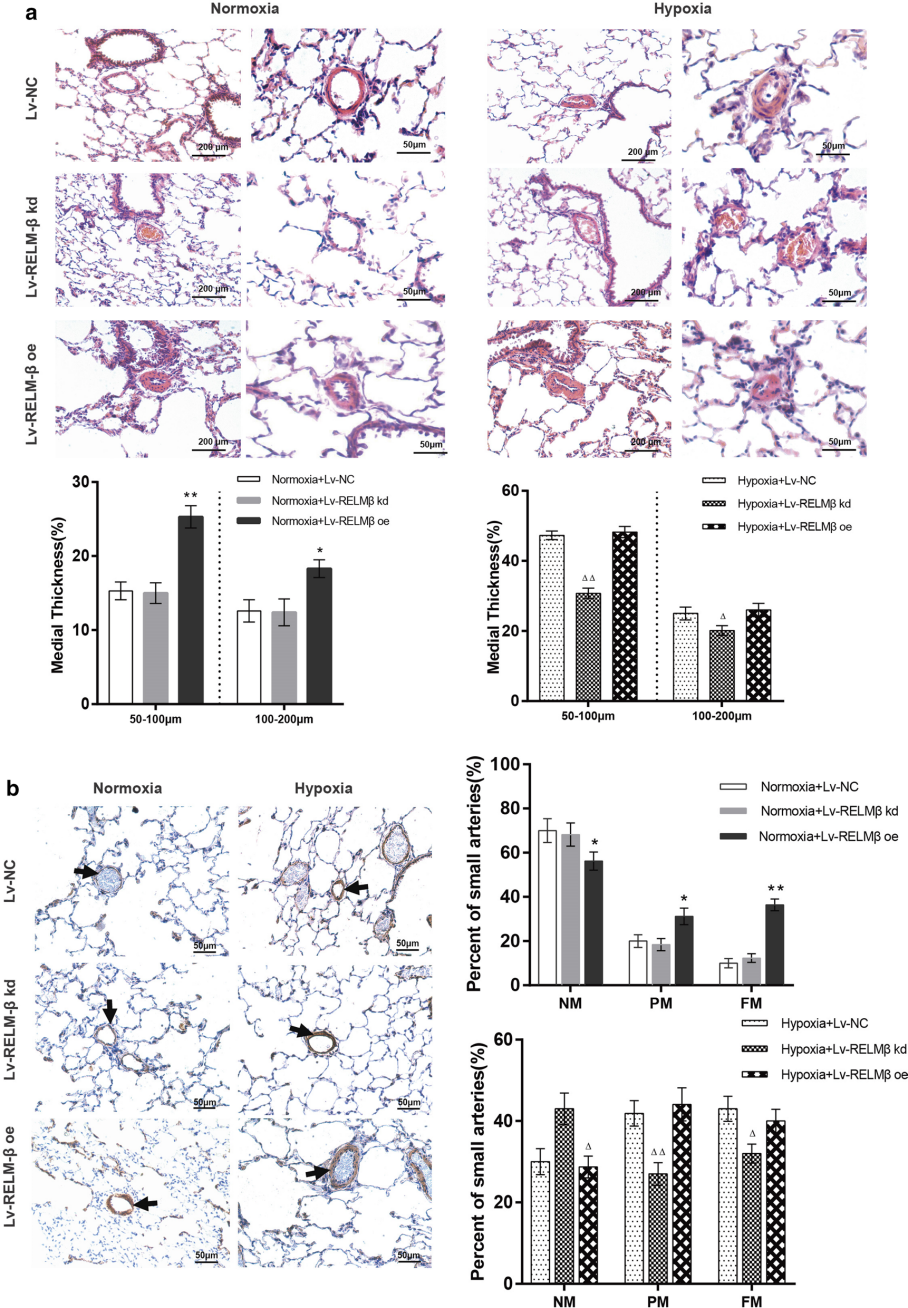
Proremodeling effect of RELM-β in pulmonary arterioles. The mean arterial wall thickness in different groups was examined by H&E staining (scale bar: 50 μm). Morphometric analysis of rat pulmonary arterioles of different external diameters after exposure to normoxia or hypoxia for 21 days in different groups (**a**). The walls of small arterioles were stained with antibodies against α-smooth muscle actin and the muscularization of small pulmonary arteries is indicated as NM, PM, and FM in rat lungs 21 days after initial intratracheal instillation of lentivirus. The mean arterial wall thickness examined by H&E staining in different groups (scale bar: 200 μm) (**b**). *NM* nonmuscular, *PM* partially muscular, *FM* fully muscular. n = 6 rats in each group; the number of vessels counted is ≥ 20 for each bar. *P < 0.05 versus the Normoxia + Lv-NC group; **P < 0.01 versus the Normoxia + Lv-NC group; ^**△**^P < 0.05 versus the Hypoxia + Lv-NC group; ^**△△**^P < 0.01 versus the Hypoxia + Lv-NC group

Compared with the control group, the Lv-RELM-β group manifested increased mRVSP (Fig. 3d), RS/LV + S (Fig. 3e), and pulmonary artery wall thickness (Fig. 4a) (P < 0.05); these effects were similar to the influence of hypoxia. However, the hypoxia plus RELM-β overexpression (oe) group did not have significant differences compared with the RELM-β overexpression under normoxia group; there were no differences between the normoxia + RELM-β kd and normoxia + NC groups and between the hypoxia + RELM-β oe and hypoxia + NC groups (P > 0.05). Moreover, RELM-β gene silencing resulted in significant attenuation of mRVSP and pulmonary artery wall thickness in the rat model of HPH (P < 0.05) (Figs. 3d and 4a).

External diameter of small arterioles (25–200 μm) was measured to estimate percentage of media thickness (%MT) and muscularization by histological staining. As shown in Fig. 4a, b, α-smooth muscle actin positive staining and %MT were increased in the normoxia + RELM-β oe group and in rats exposed to 21 days of hypoxia and substantially reduced in the hypoxia + RELM-β kd group in small (50–100 μm) (P < 0.01) and large vessels (100–200 μm) (P < 0.05). RELM-β gene silencing also substantially decreased the percentage of NM small arteries and increased the percentage of PM and FM small arteries under hypoxic conditions (P < 0.05) but there was no statistically significant difference under normoxic conditions (P > 0.05). RELM-β overexpression displayed an opposite trend and influenced small artery muscularization (P < 0.05) (Fig. 4b). These results indicate that RELM-β enhances mRVSP and associated cardiopulmonary vascular remodeling in the HPH rat model.

### Effects of RELM-β on phosphorylated PI3K, Akt, mTOR, PKC, and MAPK proteins

**Fig. 5:**
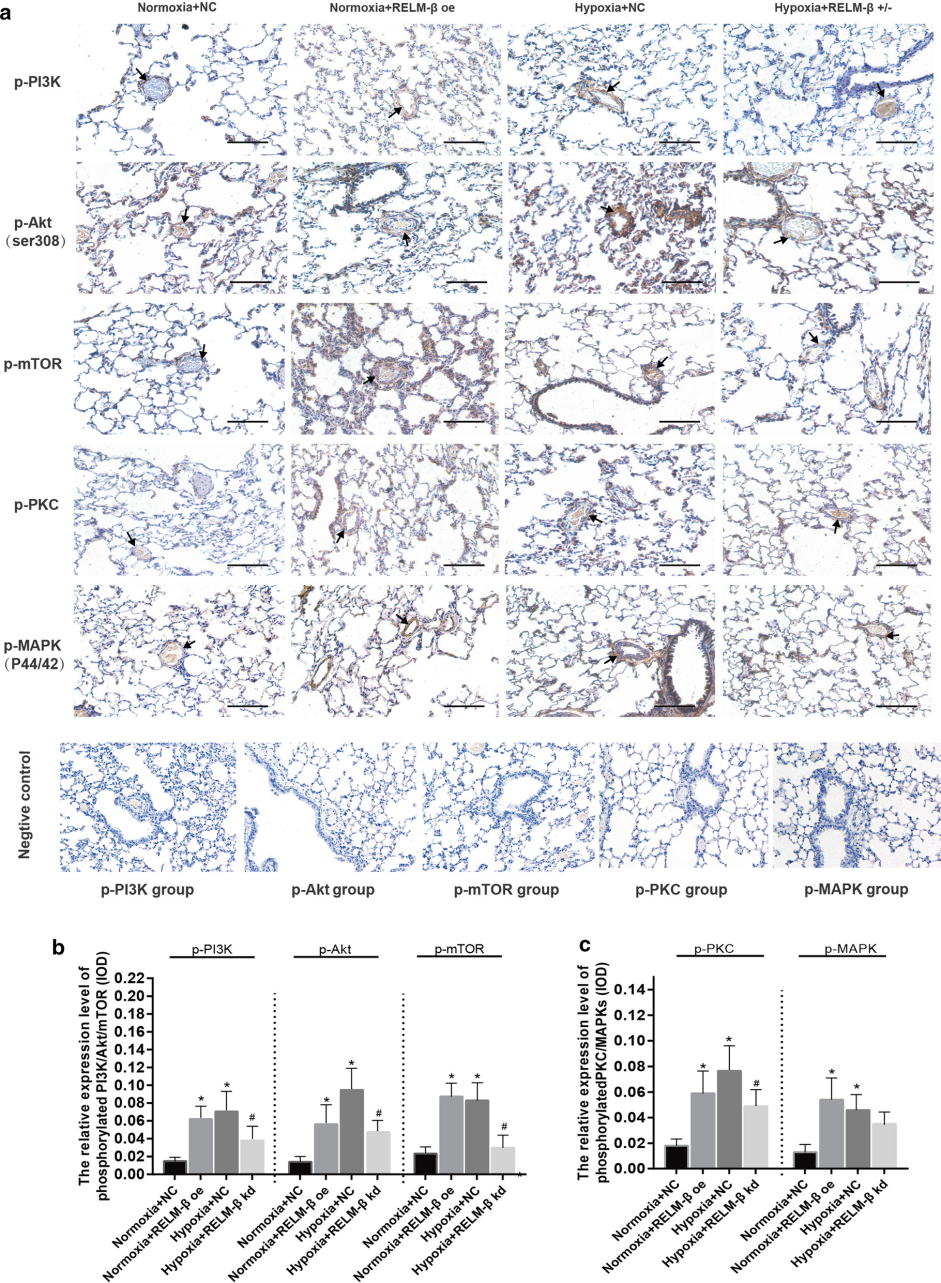
Alteration of phosphorylated PI3K/Akt/mTOR and PKC/MAPK proteins by RELM-β. Immunohistochemical analysis of phosphorylated PI3K/Akt/mTOR and PKC/MAPK proteins in lung tissue (positive signals in pulmonary arteries were used for analysis); the black arrows indicate that these phosphorylated proteins occur in remolded pulmonary arteries (scale bar: 200 μm) (**a**). The IOD value of phosphorylated PI3K/Akt/mTOR in different groups (**b**). The IOD value of phosphorylated PKC/MAPKs in different groups (**c**). n = 6 rats in each group; the number of vessels counted is ≥ 20 for each bar. *P < 0.05 versus the Normoxia + Lv-NC group. ^**#**^P < 0.05 versus the Hypoxia + Lv-NC group

Inhibition of PI3K and PKC has been shown to suppress the PASMC proliferation induced by RLEM-β; hence, we hypothesized that these signaling pathway proteins are the regulators downstream of RLEM-β in an HPH rat model. Immunohistochemistry results showed that p-PI3K, p-Akt, p-mTOR, p-PKC, and p-MAPKs levels in the media of small pulmonary arteries and lung of rats exposed to hypoxia or treated with RLEM-β overexpression vector were increased compared with those in the control groups (P < 0.05) (Fig. 5a). The relative expression levels measured as IOD values showed that the effect of hypoxia on phosphorylation of Akt, mTOR, and PKC was significantly reduced by knockdown of RLEM-β (P < 0.05) (Fig. 5b, c).

**Fig. 6:**
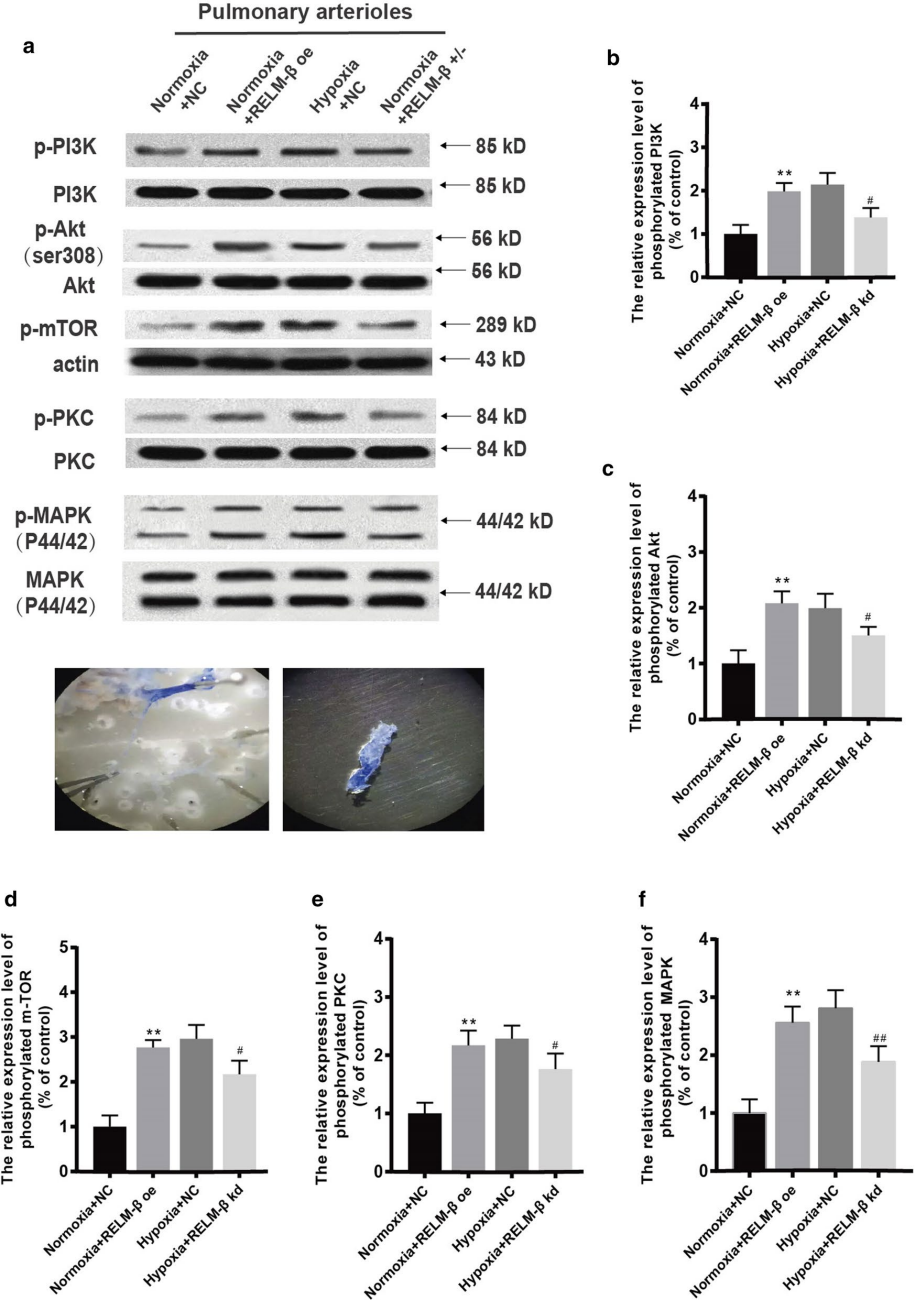
Alteration of phosphorylated PI3K/Akt/mTOR and PKC/MAPK proteins by RELM-β. The WB results of phosphorylated PI3K/Akt/mTOR and PKC/MAPK protein-isolated pulmonary arteries (**a**). The relative expression level of p-PI3K in isolated pulmonary arteries (**b**). The relative expression level of p-Akt in isolated pulmonary arteries (**c**). The relative expression level of p-mTOR in isolated pulmonary arteries (**d**). The relative expression level of p-PKC in isolated pulmonary arteries (**e**). The relative expression level of p-MAPK in isolated pulmonary arteries (**f**). n = 6 in each group. *P < 0.05 versus the Normoxia + Lv-NC group. **P < 0.01 versus the Normoxia + Lv-NC group; ^**#**^P < 0.05 versus the Hypoxia + Lv-NC group; ^**##**^P < 0.01 versus the Hypoxia + Lv-NC group

Isolated pulmonary arterioles and lung tissue homogenates were used to perform Western blot (Fig. 6a). The results of relative quantification demonstrated that the p-PI3K/PI3K ratio was considerably increased in the LV-RELM-β-treated rats compared with that in untreated rats under normoxia (P < 0.01). RELM-β knockdown significantly decreased the p-PI3K/PI3K ratio compared with that in the hypoxia + NC group (P < 0.01) (Fig. 6b). The changes in the relative levels of phosphorylated Akt (Fig. 6c), mTOR (Fig. 6d), PKC (Fig. 6e), and MAPK (p44/42) (Fig. 6f) were similar to those detected in the case of PI3K and were statistically significant (P < 0.01). Although there was no regular pattern in each group, the results indicated that RELM-β plays a similar role in phosphorylation of these signal molecules in hypoxia (see Fig. 6b–f). Overall, the evidence suggests that RELM-β may promote mRVSP increase and pulmonary vascular remodeling, at least in part, through the PI3K/Akt/mTOR and PKC/MAPKs signaling.

### RELM-β promotes proliferation and Ca^2+^ release in cultured primary hPASMCs

**Fig. 7:**
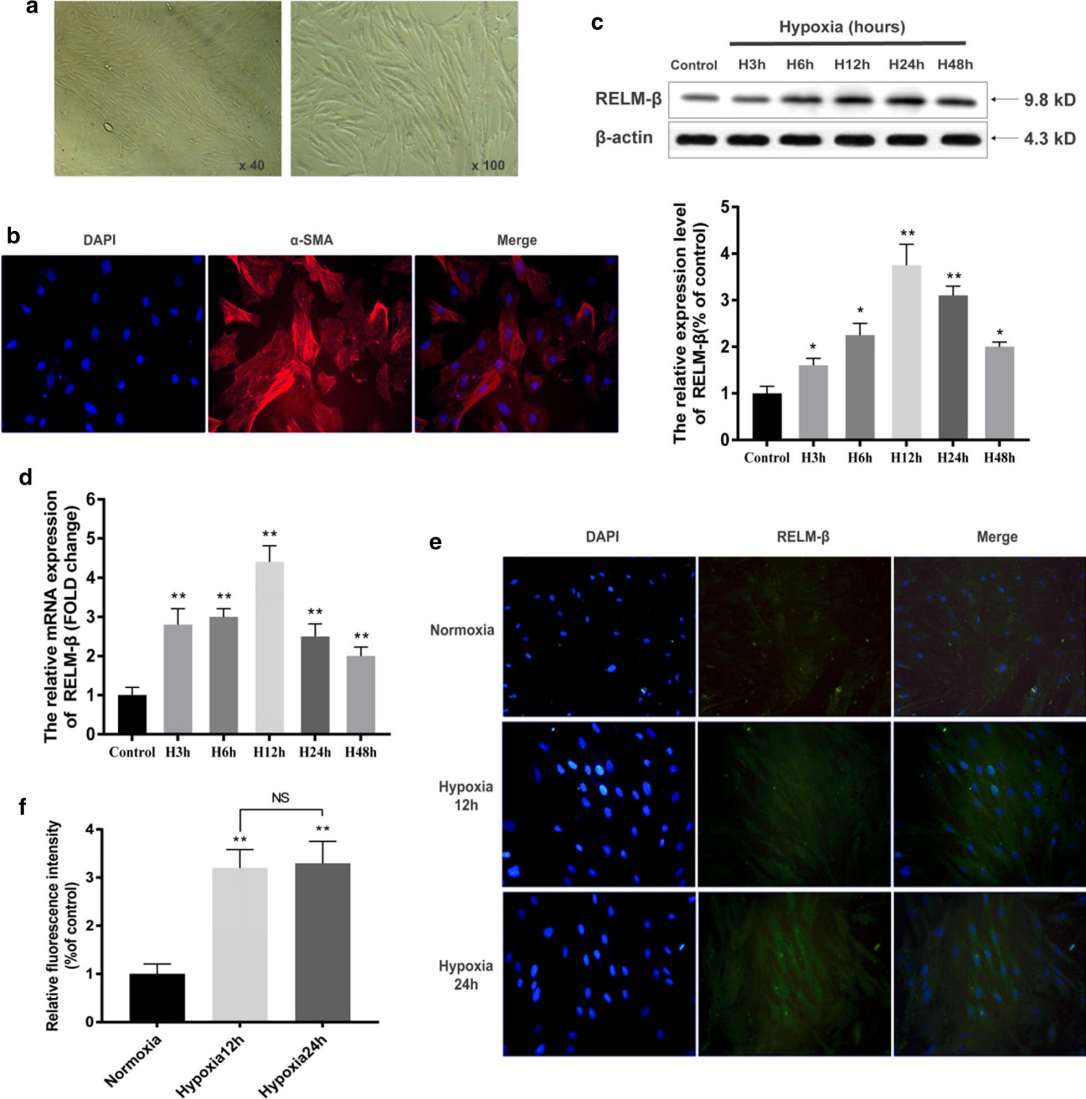
Hypoxia induces RELM-β expression in primary cultured PASMCs in a time-dependent manner. Light microscopy revealed typical "peak-valley" growth of smooth muscle cells (**a**). Immunofluorescence staining of α-SMA showed that the cytoplasm of the cells was clearly stained with red fluorescence, which was distributed along the myofilaments and arranged in parallel (**b**). WB analysis of RELM-β protein expression levels at different durations of hypoxia (3 h, 6 h, 12 h, 24 h, and 48 h) (**c**). The relative mRNA level of RELM-β at different durations of hypoxia (**d**). The results in **c**, **d** show that RELM-β expression peaked at 12 h and remained high until 48 h of hypoxia. We used immunofluorescence staining of RELM-β to verify this pattern (**e**, **f**). n = 6 in each group. *P < 0.05 versus the control group; **P < 0.01 versus the control group

Previous studies have shown that the ortholog of RELM-β can increase intracellular Ca^2+^ via extracellular calcium influx in PASMCs [16, 21]. To detect a direct effect of RELM-β on cell proliferation and Ca^2+^ signaling, primary PASMCs were cultured. Light microscopy detected typical "peak-valley" growth of smooth muscle cells. The results of α-SMA immunofluorescence staining showed that the cytoplasm of the cells has red fluorescence signal distributed along the myofilaments and arranged in parallel patterns (see Fig. 7a, b). Treatment of PASMCs with hypoxia upregulated RELM-β protein and mRNA expression in a time-dependent manner (3, 6, 12, 24, and 48 h), and the maximal levels were detected at 12 and 24 h (P < 0.01 compared with those in the control group) (see Fig. 7c–e) confirming that hypoxia induces RELM-β in PASMCs.

**Fig. 8:**
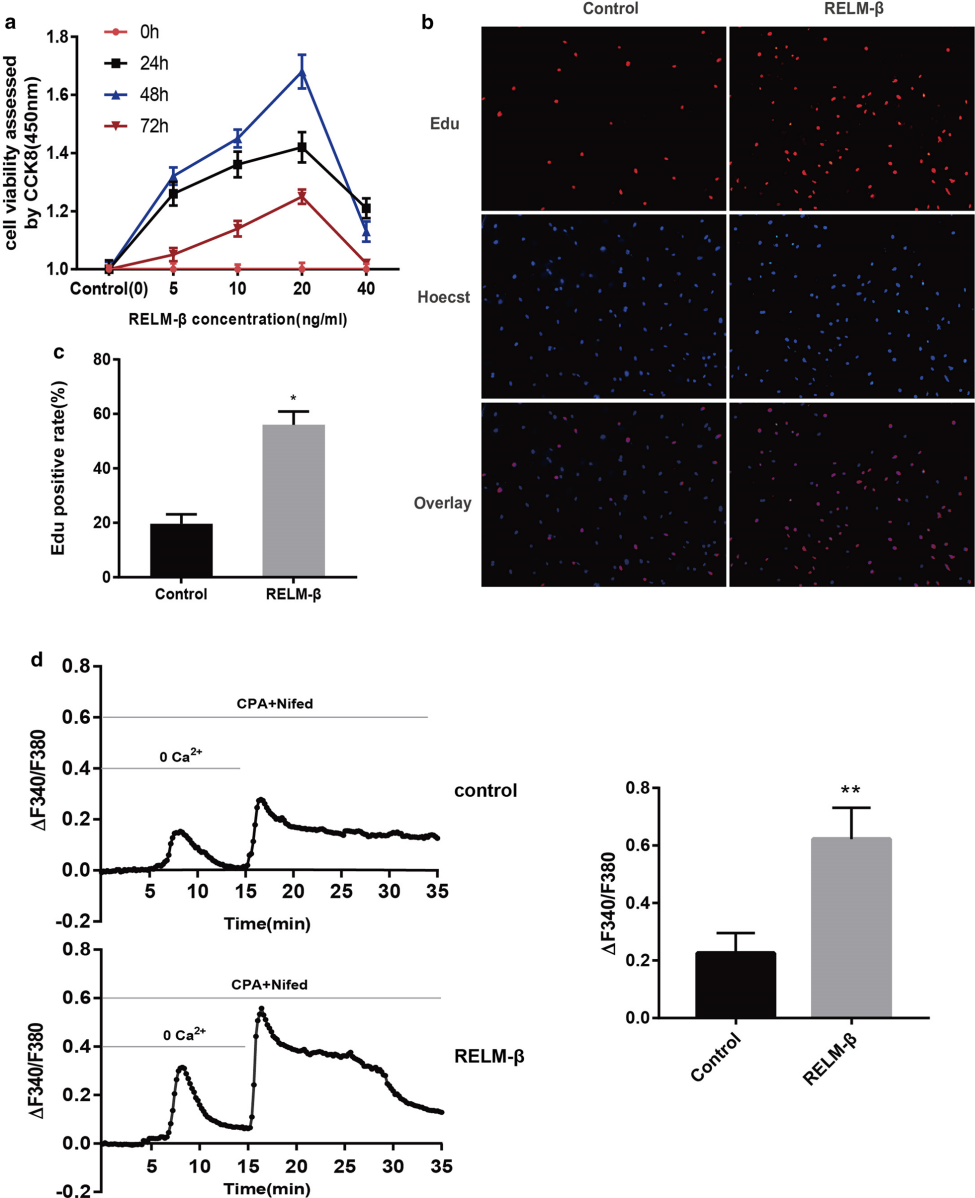
RELM-β promotes cell proliferation and Ca^2+^ release in primary cultured hPASMCs. The cell viability of hPASMCs treated with different concentrations (0 ng/ml, 5 ng/ml, 10 ng/ml, 20 ng/ml, or 40 ng/ml) of RELM-β for 0, 24, 48 or 72 h using the CCK-8 assay (**a**). The percentage of EdU-positive hPASMCs treated with 0 (control) or 20 ng/ml RELM-β for 48 h using EdU immunofluorescent staining (**b**, **c**). RELM-β enhanced the store-operated Ca^2+^ entry intracellular Ca^2+^ concentration ([Ca^2+^]_i_) in hPASMCs. RELM-β (20 ng/ml for 48 h) enhanced SOCE in hPASMCs. Time course of Δ[Ca^2+^]_i_ (ΔF340/F380) before and after the restoration of extracellular Ca^2+^ to 2.5 mM in control hPASMCs (top) and RELM-β-treated (20 ng/ml for 48 h) hPASMCs (bottom) perfused with Ca^2+^-free Krebs–Ringer bicarbonate solution (KRS) containing 10 μM cyclopiazonic acid (CPA) and 5 μM nifedipine. Ca^2+^-free KRS also contained 1 mM EGTA to chelate any residual Ca^2+^. The average peak change in Δ[Ca^2+^]_i_ (ΔF340/F380) after restoration of extracellular Ca^2+^(SOCE) in control and RELM-β-treated hPASMCs (right) (**d**). n = 3 in each group. *P < 0.05 versus control group; **P < 0.01 versus control group

Then, PASMCs were treated with various concentrations of recombinant RELM-β for 0, 24, 48, and 72 h. The CCK-8 assay showed that cell viability was the highest at 48 h at each concentration of RELM-β, and 20 ng/ml RELM-β had the maximum effect (Fig. 8a). The EdU assay results confirmed that treatment of hPASMCs with 20 ng/ml RELM-β for 48 h promotes cell proliferation (P < 0.01) (Fig. 8b). To determine whether RELM-β-induced hPASMCs proliferation is associated with SOCE, [Ca^2+^]_i_ were monitored by fura-2 fluorescence imaging (340/380 nm ratio, F340/F380). As shown in Fig. 8d, SOCE activated by depletion of Ca^2+^ stores with CPA significantly increased in hPASMCs treated by 20 ng/ml RELM-β for 48 h (0.62 ± 0.06 for RELM-β treatment cells, and 0.23 ± 0.04 for control cells; P < 0.05). Our results indicate that RELM-β promotes the proliferation and Ca^2+^ influx of hPASMCs.

### Ca^2+^ is involved in the activation of the PI3K/Akt/mTOR and PKC/MAPK pathways and PASMC proliferation stimulated by RELM-β

To investigate whether Ca^2+^ plays a role in hPASMC proliferation induced by RELM-β and is an upstream regulator of the PI3K/Akt/mTOR or PKC/MAPK signaling pathways, cells were pretreated with the intracellular Ca^2+^ antagonist BAPTA-AM (10 μM) or the extracellular Ca^2+^ antagonist EGTA (4 mM) with 0 mM Ca^2+^ in extracellular media and were then treated with 20 ng/ml RELM-β for 48 h.

**Fig. 9:**
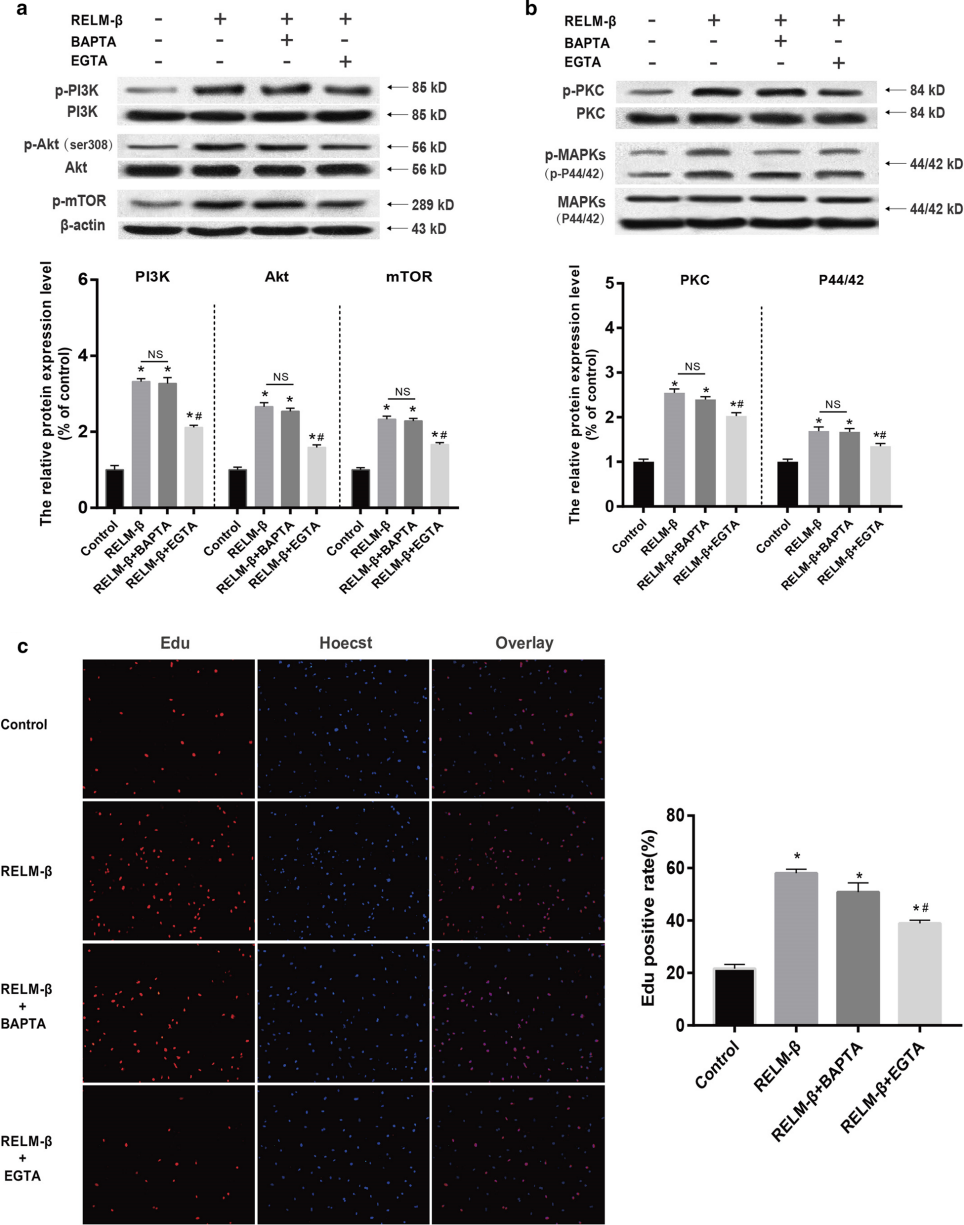
Ca^2+^ is involved in the activation of the PI3K/Akt/mTOR and PKC/MAPK pathways and PASMC proliferation by RELM-β stimulation. The WB results and relative p-PI3K, p-Akt, and p-mTOR protein levels of PASMCs treated with 0 (control) or 20 ng/ml RELM**-**β, RELM**-**β + 10 μM BAPTA-AM, or RELM**-**β + 4 mM EGTA (with 0 mM extracellular Ca^2+^) (**a**). The WB results and relative p-PKC and p-MAPK protein levels of PASMCs treated with 0 (control) or 20 ng/ml RELM**-**β, RELM**-**β + BAPTA, or RELM**-**β + EGTA for 48 h (**b**). The percentage of EdU-positive PASMCs treated with 0 (control) or 20 ng/ml RELM-β, RELM**-**β + BAPTA-AM, or RELM**-**β + EGTA for 48 h using EdU immunofluorescent staining (**c**). n = 3 in each group. *P < 0.05 versus the control group; ^**#**^P < 0.05 versus the RELM**-**β group

The Western blot results showed that the levels of phosphorylated PI3K, Akt, mTOR, PKC. And MAPK proteins were significantly increased by RELM-β stimulation (P < 0.01) (see Fig. 9a, b). EGTA treatment significantly inhibited the phosphorylation of these proteins compared with that in the RELM-β group (P < 0.01). However, BAPTA treatment did not inhibit PI3K, Akt, mTOR, PKC, or MAPK phosphorylation (P > 0.05) (see Fig. 7a, b). As shown in Fig. 7c, cell proliferation induced by RELM-β was suppressed by two Ca^2+^ chelators. Additionally, the RELM-β + EGTA group had a 12% lower proliferation assessed by the EdU assay compared with that in the RELM-β + BAPTA-AM group (P < 0.05). This decrease may result from preferential stimulation of extracellular Ca^2+^ influx and not intracellular Ca^2+^ release by RELM-β. This result suggests that Ca^2+^ is essential for RELM-β-induced activation of the PI3K/Akt/mTOR or PKC/MAPK signals and PASMC proliferation.

### Involvement of PI3K/Akt/mTOR and PKC/MAPKs in RELM-β-induced PASMC proliferation

**Fig. 10:**
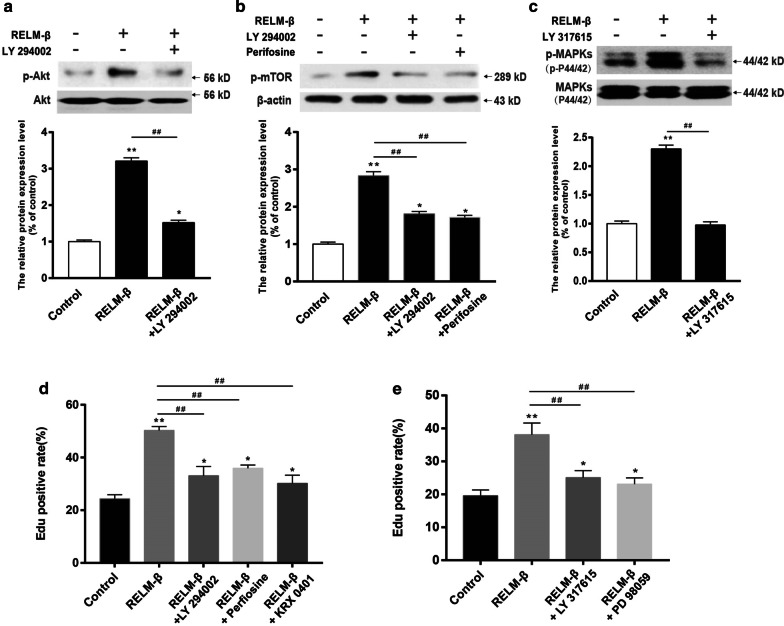
Involvement of PI3K/Akt/mTOR and PKC/MAPKs in RELM-β-induced PASMC proliferation. The WB results and relative p-Akt protein levels of PASMCs treated with 0 (control) or 20 ng/ml RELM-β or RELM-β + LY294002 (10 μM, a PI3K inhibitor) (**a**). The WB results and relative p-mTOR protein levels of PASMCs treated with 0 (control) or 20 ng/ml RELM-β, RELM-β + LY294002 and RELM-β + perifosine (100 nM, an Akt inhibitor) (**b**). The WB results and relative p-MAPK protein levels of PASMCs treated with 0 (control) or 20 ng/ml RELM-β or RELM-β + LY317615 (10 μM, a PKC inhibitor) (**c**). The percentage of EdU-positive PASMCs treated with 0 (control) or 20 ng/ml RELM-β, RELM**-**β + LY294002, RELM**-**β + perifosine, or RELM**-**β + rapamycin (100 nM, a mTOR inhibitor) (**d**) and with RELM-β + LY317615 or RELM**-**β + PD98059 (20 μM, a MAPK inhibitor) (**e**) for 48 h using EdU immunofluorescence staining (**c**). n = 3 in each group. *P < 0.05 versus the control group; **P < 0.01 versus the control group; ^**##**^P < 0.01 between two groups indicated by a line

Our previous results and the data of others authors [14, 29] have shown that the PI3K and PKC signaling pathways are downstream of RELM-β in the hypoxia-induced PASMC proliferation, To determine the role and mechanism of action of RELM-β in vitro, the levels of phosphorylated proteins of each signaling component were assessed by Western blot analysis. As shown in Fig. 10a, b, phosphorylated Akt and mTOR levels were significantly suppressed by a PI3K inhibitor LY294002 compared with those detected in the recombinant RELM-β group (P < 0.01) but remained higher than those in the control group (P < 0.05). Levels of phosphorylated mTOR protein were also decreased by pretreatment of the cells with an Akt inhibitor perifosine (P < 0.01). Akt and mTOR are downstream of PI3K, and mTOR is downstream of Akt. As shown in Fig. 10c, the level of phosphorylated p-MAPK in the group treated with a PKC inhibitor LY317615 was statistically significantly different from that in the RELM-β group (P < 0.01) indicating that MAPKs are the downstream effectors of PKC.

The effects of PI3K/Akt/mTOR and PKC/MAPKs on RELM-β-induced PASMC proliferation were also investigated using the EdU assay. As shown in Fig. 10d, e, the proliferation of PASMCs was significantly suppressed in the PI3K inhibitor, Akt inhibitor, mTOR inhibitor, PKC inhibitor, and MAPK inhibitor groups compared to that in the RELM-β group (P < 0.01). Finally, our results showed that RELM-β induces PASMC proliferation via the PI3K/Akt/mTOR and PKC/MAPK signaling pathways.

## Discussion

Hypoxia-induced pulmonary hypertension (HPH) is a severe complication of advanced COPD. The disease is characterized by abnormal PASMC proliferation and subsequent pulmonary artery remodeling. We initially focused on RELM-β because of its mitogenic effect on several types of lung cells and especially on PASMCs [12, 14, 29] and its high expression in the pulmonary artery of patients with PAH [14]. The results of the present study demonstrate that RELM-β is endogenously expressed in vivo and in vitro after hypoxic stimulation. RELM-β significantly promotes PASMC proliferation and pulmonary vascular remodeling in response to hypoxia. The proliferative effect of RELM-β is apparently related to the Ca^2+^, PI3K/Akt/mTOR, and PKC/MAPK signaling pathways. To the best of our knowledge, this is the first report describing the role of RELM-β in HPH and increase in [Ca^2+^]_i_ in hPASMCs.

RELM-β belongs to a highly conserved secretory protein family (RELMs) that includes two key members: RELM-α (FIZZ1/HIMF) and RELM-β (FIZZ2) [7]. RELM is a newly defined protein family discovered by Holcomb during the investigation of airway inflammation in allergic asthma [30]. RELM-β is inherently expressed in colonic epithelium, and its expression can be upregulated to a high level in airway smooth muscle or lung tissue by an allergen [27, 31] or bleomycin [13, 14] and in lung fibroblasts, macrophages, and SMCs by hypoxia [12, 32]. The results of the current study indicate that chronic hypoxia induces endogenous RELM-β in the lung of rats in a HPH model at the mRNA or protein level. Strong RELM-β immunostaining is colocalized with α-SMA in the remodeling pulmonary arteries, which is in agreement with the data on the location of RELM-β in PAH patients [14]. This expression pattern is similar to that of RELM-α in an HPH rat model [24]. Additionally, the level of RELM-β in BALF is decreased after 7 days of hypoxia, which is consistent with the trends observed in the serum and lung. This decrease may be because chronic hypoxia mainly increases RELM-β in the pulmonary vasculature rather than in alveolar epithelium (see Fig. 2d), and higher level of RELM-β is secreted into the circumvasculature and lumen. Consistent with the in vivo results, the data in hPASMCs showed that hypoxia upregulates RELM-β in a time-dependent manner. These data indicate that RELM-β plays a role in HPH through the autocrine and paracrine mechanisms.

Similar to other cytokines participating in PASMC proliferation, such as TGF-β and VEGF-1 [33, 34], Angelini et al. [14] demonstrated that treatment with 100 ng/ml recombinant human RELM-β (rhRELM-β) for 48 h significantly increased the proliferation of hPASMCs and human lung microvascular endothelial cells (HMVECs). Renigunta et al. [12] also demonstrated that hPASMCs transfected with RELM-β have higher proliferation compared with that of the wild-type cells. In the present study, our result showd that rhRELM-β promoted hPASMC proliferation in a dose-dependent manner. To further investigate the mitogenic effect of RELM-β in HPH, two approaches were used: overexpression of RELM-β in the lungs of normal rats and blockade of RELM-β in a chronic hypoxia model of PH. First, we showed that overexpression of RELM-β induces vascular remodeling and hemodynamic changes identical to those associated with chronic hypoxia-induced PH and other forms of PH. Second, in vivo knockdown of RELM-β attenuates vascular remodeling and hemodynamic changes associated with PH. The neo-muscularization of small non-muscular pulmonary arterioles (external diameter < 200 μm) is the main source of pulmonary vascular resistance and the hallmark of HPH [35, 36]. Then, we showed that RELM-β gene transfer into the pulmonary tissue induces media thickening and increases the proportion of partially muscular and fully muscular arterioles. RELM-β knockdown has an opposite effect. These results suggest that RELM-β directly enhances proliferation and plays an important role in the development of PH, which is completely consistent with that of RELM-α [24].

RELM-α is the first member of the RELM family identified in the studies of pulmonary diseases in rodents. Humans lack the RELM-α gene, but RELM-β shares 69% cDNA sequence identity with RELM-α, and their homology in the amino acid sequence is as high as 58.6% [8]. Additionally, the expression pattern and function of the two molecules are very similar. Therefore, RELM-β is considered a direct functional homolog of RELM-α in humans. Teng et al. [9, 37] detected a hypoxia inducible factor (HIF)-binding site in the 3′ region of the RELM-α gene promoter. This finding may explain the regulatory effect of hypoxia on RELM-α and RELM-β. A previous study demonstrated that RELM-α induces intracellular calcium release via the PLC-IP_3_ pathway [16]. A recent study found that RELM-α has a calcium-binding site called CaSR (Ca^2+^-sensing receptor) and induces Ca^2+^ influx in a SOCE (store-operated calcium entry)-dependent manner [21, 38, 39]. Intracellular free calcium ([Ca^2+^]_i_) is a known messenger involved in cell proliferation [40]. Lee et al. [19] demonstrated that hypoxia upregulates the Ca^2+^ concentration in chicken hepatocytes. [Ca^2+^]_i_ enhances the PI3K, Akt, mTOR, PKC, and MAPK phosphorylation and activates cyclin E and D1 and CDK 2 and 4 signaling ultimately promoting the cells into the mitotic phase [20]. RELM-β activates PI3K/AKT and ERK/MAPK in the airway smooth muscle cells, induces the expression of TGF-β, VEGF, and VEGFR, and promotes cell proliferation [29, 32]. Renigunta et al. [12] showed that the proliferative effect of RELM-β can be suppressed by two inhibitors of PI3K (phosphatidylinositol 3-kinase) and protein kinase C (PKC). Moreover, RELM-β can activate the Ca^2+^/ERK/PKC signaling in goblet cells [41]. Thus, these molecular mechanisms were investigated in the present study.

Our results showed that 20 ng/ml RELM-β has a stimulatory effect on the intracellular Ca^2+^ concentration in PASMCs. Treatment with CPA and nifedipine indicated that SOCE is involved in the process. Then, intracellular and extracellular Ca^2+^ blockers were used. The results showed that the stimulatory effect of RELM-β on phosphorylation of PI3K, Akt, mTOR, PKC, and MAPK is significantly suppressed by EGTA. The PASMC proliferation was also decreased by *extracellular* Ca^2+^ *chelation*. The results indicate that RELM-β induces hPASMCs proliferation at least partly through the Ca^2+^ influx. PI3K/Akt/mTOR and PKC/MAPKs are probably downstream of Ca^2+^. To confirm the role and the relationship of PI3K/Akt/mTOR and PKC/MAPK, each pathway component was inhibited; the data indicated that these signaling pathways participate in PASMC proliferation induced by RELM-β. The results are highly consistent with our in vivo study; the phosphorylated PI3K, Akt, mTOR, PKC, and MAPK levels are considerably increased by RELM-β overexpression in the pulmonary arterioles similar to those detected in hypoxia. RELM-β gene silencing has an opposite effect. These findings demonstrate the role of RELM-β in hPASMC proliferation and HPH and suggest molecular mechanisms of action.

The major limitations of this study include the following: (1) We used a gene silencing animal model rather than a gene knockout model. RELM-α plays a crucial role in rat embryonic lung development [42]; hence, we were not sure whether RELM-β knockout causes early death of the animals. (2) The effect of RELM-β on Ca^2+^ influx is merely an observation, and the exact mechanism of Ca^2+^ release induced by RELM-β remains to be investigated. A conditional gene knockout model may be developed in the subsequent studies to investigate the function of RELM-β in HPH. Identification of a potential CaSR-binding site of RELM-β and the upstream molecules of RELM-β requires further investigation.

## Conclusion

Thus, our study demonstrated that RELM-β is induced in hypoxia and has a mitogenic effect in HPH. This effect was observed in a rat model of HPH and cultured primary hPASMCs. These results suggest that RELM-β may induce PASMC proliferation and pulmonary artery remodeling leading to HPH at least partially through the PI3K/Akt/mTOR and PKC/MAPK pathways. The phosphorylation of these molecules is apparently activated by Ca^2+^ influx. Our findings contribute to the understanding of the pathophysiology of RELM-β in HPH and assist in the development of novel therapies for the disease.

## Data Availability

All data generated or analyzed during this study are included in this published article and its supplementary information files. The datasets used and/or analyzed during the current study are available from the corresponding author on reasonable request.

## Abbreviations

PAH: Pulmonary arterial hypertension
HPH: Hypoxia-induced pulmonary hypertension
PASMCs: Pulmonary arterial smooth muscle cells
mPAP: Mean pulmonary arterial pressure
mRVSP: Mean right ventricular systolic pressure
RVHI: Right ventricular hypertrophy index
BALF: Bronchoalveolar lavage fluid
